# Japanese Encephalitis Virus Induce Immuno-Competency in Neural Stem/Progenitor Cells

**DOI:** 10.1371/journal.pone.0008134

**Published:** 2009-12-02

**Authors:** Sulagna Das, Debapriya Ghosh, Anirban Basu

**Affiliations:** National Brain Research Centre, Manesar, Haryana, India; Institut Pasteur, France

## Abstract

**Background:**

The low immunogenicity of neural stem/progenitor cells (NSPCs) coupled with negligible expression of MHC antigens has popularized their use in transplantation medicine. However, in an inflammatory environment, the NSPCs express costimulatory molecules and MHC antigens, and also exhibit certain immunomodulatory functions. Since NSPCs are the cellular targets in a number of virus infections both during postnatal and adult stages, we wanted to investigate the immunological properties of these stem cells in response to viral pathogen.

**Methodology/Principal Findings:**

We utilized both *in vivo* mouse model and *in vitro* neurosphere model of Japanese encephalitis virus (JEV) infection for the study. The NSPCs residing in the subventricular zone of the infected brains showed prominent expression of MHC-I and costimulatory molecules CD40, CD80, and CD86. Using Flow cytometry and fluorescence microscopy, we observed increased surface expression of co-stimulatory molecule and MHC class I antigen in NSPCs upon progressive JEV infection *in vitro*. Moreover, significant production of pro-inflammatory cyto/chemokines was detected in JEV infected NSPCs by Cytokine Bead Array analysis. Interestingly, NSPCs were capable of providing functional costimulation to allogenic T cells and JEV infection resulted in increased proliferation of allogenic T cells, as detected by Mixed Lymphocyte reaction and CFSE experiments. We also report IL-2 production by NSPCs upon JEV infection, which possibly provides mitogenic signals to T cells and trigger their proliferation.

**Conclusion/Significance:**

The *in vivo* and *in vitro* findings clearly indicate the development of immunogenicity in NSPCs following progressive JEV infection, in our case, JEV infection. Following a neurotropic virus infection, NSPCs possibly behave as immunogenic cells and contribute to both the innate and adaptive immune axes. The newly discovered immunological properties of NSPCs may have implications in assigning a new role of these cells as non-professional antigen presenting cells in the central nervous system.

## Introduction

The role of neural stem/progenitor cells (NSPCs) in brain repair and regeneration has been well documented [1], [2]. NSPCs are a self-renewing, multi-potential population of cells which are capable of differentiating into neurons, astrocytes and oligodendrocytes. Usually housed in specific neurogenic areas of the developing and the adult brain, namely Subventricular zone (SVZ) and hippocampus, these cells function in brain development, memory formation as well as brain repair [3]. The regenerative potential of NSPCs has been used extensively for transplantation therapy in a number of brain disorders. Transplantation with NSPCs have ameliorated the clinical features in a range of experimental models of neurological disease, including stroke [4]; Parkinson's disease [5]; spinal cord trauma [6]; and multiple sclerosis [7], [8]. An important characteristic feature of NSPCs that have helped to overcome the challenges of transplantation is the low cell surface expression of Major Histocompatibility Complex (MHC) gene products. Both T-cells and Natural killer (NK) cells of the host can recognize the foreign progenitors by the surface MHC class I expression and reject the grafted cells. Many researchers have demonstrated absence or negligible expression of MHC class I and II antigen by early and later passaged neurospheres, which protects these cells from immune recognition [9]. The low immunogenicity of NSPCs [10] coupled with their low expression of MHC class I and II have popularized their use in transplantation medicine [11], [12].

In recent years, the immunological properties of NSPCs and how different exogenous factors influence their immunogenicity have begun to be explored. Previous reports indicate that the isolated NSPCs express costimulatory molecules, like CD80 (B7.1) and CD86 (B7.2), though their exact functions have not been elucidated. Exposure of NSPCs to an inflammatory environment triggers the expression of these costimulatory molecules like increased CD80 expression in the SVZ during the course of EAE [13]. Moreover, enhanced expression of CD80 and CD86, as well as MHC class I (but not MHC class II), is observed *in vitro* upon exposure of NSPCs to pro-inflammatory cytokines like IFN-γ and TNF-α (the prototypical Th1 cytokines) [13], [14]. The functional relevance of enhanced expression of costimulatory and MHC proteins on NSPCs was shown in the ability of these cells to costimulate T-cells and trigger their proliferation [13], [15]. In an inflammatory milieu, T-cells can interact with NSPCs *in vitro*, but whether such an interaction occurs *in vivo* is still unknown.

The role of NSPCs in immunomodulation, i.e. in regulating the immune functions of other immune effector cells has been established in recent years. Several reports indicate that NSPCs can inhibit inflammatory process in cases of chronic recurrent CNS inflammation like autoimmune diseases MS/EAE. Transplanted NSPCs are able to persist in chronic inflamed environment in the areas of brain damage, by means of cross-talk with the infiltrating T-cells and the peripheral immune system [16], [17]. The immunomodulatory functions of human NSPCs have also been shown in conditions of spinal cord injury, acute and chronic EAE (animal models of MS) and neurodegenerative conditions [18].

Considering the immunogenic role of NSPCs and the immune-regulation that they exert, we wanted to investigate how these cells would behave in neurotropic virus infections. Most viruses infecting the CNS cause acute neuroinflammation, characterized by astrocytic and microglial activation and release of pro-inflammatory molecules [19], [20]. While research on the role of inflammatory stimuli in triggering immunogenicity in NSPCs has been underway, however, the immunological response of these cells following an acute viral attack in the CNS has never been explored. The CNS normally is in an immunologically quiescent state, due to deficient expression of MHC molecules, and limited entry of infiltrating T-cells into the brain parenchyma. However, following infection with RNA viruses, induction of both innate and adaptive immune response occur within the CNS [21]. Infection in neurons or other CNS cell types leads to rapid local production of type I Interferons (IFN) in a bid to curtail virus spread and replication. The damaged and infected neurons also secrete an array of cytochemokines, which in turn activate the resident CNS immune cells, astrocytes and microglia [22]. Astrocytic and microglial activation results in an inflammatory surge, and constitutes the non-specific innate immune response to virus infections. The virus-specific adaptive immune response follows, accompanied by the entry of circulating leukocytes into the CNS. Trans-endothelial migration of activated T-cells occurs and they are retained in the CNS when the viral antigen along with the appropriate MHC molecule is presented.

Emerging evidences have identified NSPC population as a potential target of neurotropic viruses, and many of these viruses establish latent infection in these cells. DNA viruses like Cytomegalovirus, RNA viruses like Lymphocytic Choriomeningitis virus (LCMV), Coxsackievirus, and Japanese encephalitis virus (JEV) and Retroviruses like HIV are examples of such viruses showing tropism for NSPCs. It is therefore imperative to study the immunological response of NSPCs following viral infection and whether these cells are able to establish a cross talk with the innate and adaptive immune axis of the host defense mechanism. This study for the first time have investigated the immunological properties of NSPCs following a virus infection (JEV infection) and have hypothesized how these cells may signal to T-cells for development of a potent immune response against virus infection.

## Materials and Methods

### Ethics Statement

All animals were handled in strict accordance with good animal practice as defined by Institutional Animal and Ethics Committee of National Brain Research Center (NBRC/IAEC/2008/41), according to the guidelines set by Committee for the Purpose of Control and Supervision of Experiments on Animals **(**CPCSEA**)**, Ministry of Environment and Forestry, Government of India.

### Virus Generation and Infection in Animals

The GP78 strain of JEV was propagated in suckling BALB/c mice and their brains were harvested when symptoms of sickness were observed. A 10% tissue suspension was made in minimum essential medium (MEM), followed by centrifugation at 10,000X g and finally filtered through a 0.22 µm sterile filter. The titration of virus particles was done by plaque formation using PS (Porcine stable Kidney) cell line [23]. Briefly, PS cell monolayers were incubated with 10-fold dilutions of the virus made in MEM containing 1% FBS for 1 hour at 37°C. After removal of viral inoculum, monolayers were overlaid with MEM containing 4% FBS, 1% low melting point agarose and a cocktail of antibiotic-antimyotic solution. Plates were incubated at 37°C for 72 hours till plaques were visible. The plaques were then counted after fixing the cells with 10% formaldehyde and staining with crystal violet [23], [24].

Suckling BALB/c mice of either sex were injected intra-cerebrally with ∼100 p.f.u (in 30 µL of PBS) of virus, and control animals received the same amount of PBS. From third day post infection (dpi), animals showed symptoms of JE including limb paralysis, poor pain response, and whole body tremor. On 4 dpi, all animals succumbed to infection.

### Neurosphere Generation and Virus Infection

BALB/c mouse pups (postnatal day 7) were decapitated and SVZ was dissected out aseptically in PGM buffer (Phosphate buffer with 1 mM MgCl_2_ and 0.6% glucose) [25]. The tissue was then dissociated in a solution of 2 mg/ml Papain with 50 µg/ml DNaseI at 37°C for 10 mins and neutralized with DMEM containing 10% FBS. Following two washes with DMEM containing 10% FBS, the cell pellet was resuspended Dulbecco's modified eagles Media/F12 (DMEM- F12) containing B27 supplement and 50 µg/ml gentamycin (all from Gibco, Carlsbad, CA). The suspension was passed through 40 µm screen and then centrifuged at 300 g for 6 mins. The cells were plated at density of 3×10^4^ cells/cm^2^ in DMEM F12 containing B27 and gentamycin, supplemented with 20 mg/ml EGF (Epidermal Growth Factor) and 10 mg/ml FGF (Fibroblast Growth Factor; R&D Systems, Minneapolis, MN). Fresh media was added after every 2 days. All *in vitro* experiments with neurospheres were carried out after minimum 2 passages and under cell density of 1.5×10^6^ cells/100 mm in petridishes.

The dissociated NSPCs after one day growth in suspension were infected with JEV at MOI = 5 (Multiplicity of Infection) for 1 hour at 37°C, washed by centrifugation, and cultured with fresh media supplemented with growth factors. Uninfected (control) and JEV infected neurospheres were collected at 1 day to 4 day post infection and subsequently processed for Flow cytometry or other co-culture experiments.

### Immunohistochemistry and Immunocytochemistry

Cryosections (20 microns) were obtained from brains of JEV infected animals and age matched controls. Fluorescence immunohistochemistry was performed on the sections for the following antibodies: anti-CD40, anti-CD80, anti-CD86, anti-H-2k^d^, (all 1∶100, BD Biosciences, San Jose, CA), and anti-Nestin (1∶250; Chemicon, Temecula, CA). For all costimulatory and MHC molecules, rat IgG2a_κ_ (1∶100) was used as isotype control, whereas for Nestin, normal mouse serum (1∶250) was used. Antigen retrieval using Antigen Unmasking solution (Vector Labs, Burlingame, CA) was done, followed by blocking with appropriate serum for 2 hours. All antibodies were incubated overnight at 4°C in a humid chamber. Respective secondary antibodies were used- anti-rat Biotinylated followed by Streptavidin-FITC (1∶250, Vector Labs) for costimulatory molecules and MHC-I, and anti-mouse Alexa Fluor 594 (1∶1000, Molecular Probes, Eugene, OR) for Nestin.

The grown neurospheres were plated on PDL (Poly D-lysine) coated chamber slides and allowed to adhere for 3 hours. The spheres were fixed in 4% formaldehyde for 20 mins at RT, following which they were incubated in blocking solution for 1 hr at room temperature (RT). They were then stained for anti-CD40, anti-CD80, anti-CD86, anti-H-2k^d^ (all 1∶100, BD Biosciences), and finally mounted with DAPI. Images were obtained using LSM 510 confocal microscope and Zeiss Apotome microscope (Zeiss, Germany).

### Cytokine Bead Array (CBA)

Cell lysates of control and JEV infected neurospheres were isolated using previously described protocol [26]. 50 µl of bead mix from Mouse Inflammation CBA kit (BD Biosciences) and 50 µl of cell lysate were incubated together for 2 hours at RT in dark. The beads were then washed and resuspended in 300 µl of Wash buffer. The beads were acquired using Cell Quest Pro Software in FACS Calibur and analyzed using BD CBA software (Becton Dickinson, San Diego, CA). Standard curve was prepared by incubating 50 µl of standard with 50 µl of bead mix.

### Mixed Lymphocyte Reaction

Splenocytes were obtained from C57BL/6 mice and enriched for T-cells by magnetic bead separation using T-cell enrichment set according to manufacturer's instructions (BD Biosciences). The purity of T-cells was tested by CD3 staining in Flow cytometry (>90% CD3+ purity). Control (uninfected) and JEV infected NSPCs (3 dpi) were treated with mitomycin C (500 µg/ml) for 4 hours and washed twice with PBS. These mitomycin treated NSPCs, either control or JEV infected were used as stimulators in the mixed lymphocyte reaction. The purified splenic T cells were used as the responders. 1×10^5^ cells of mitomycin treated NSPCs were incubated with 2×10^5^ purified splenic T-cells in 96-well plates in RPMI media supplemented with 10% FBS, 2 mM Glutamine and 500 µM β-ME. The co-culture experiments were maintained at 37°C and 5% CO_2_ for 72–80 hours. MTS reagent (Promega, Madison, USA) was then added (20 ul in each well) and following incubation for 2–3 hours, absorbance was measured at 490 nm. These experiments were performed in triplicate. Data were represented as stimulator index, calculated as the mean reading of triplicate wells stimulated with stimulator NSPCs, divided by the mean reading of triplicate wells stimulated with medium [27].

### Cell Division Analysis Using CFSE Staining

The purified splenic T cells were labeled with 5-(and 6-) carboxyfluorescein diacetate succinimidyl ester (CFSE; Molecular Probes) at a concentration of 5 µM for 8 mins in 1% BSA containing PBS at 37°C [28]. The cells were washed several times with serum containing media, and then tested for cell viability using Trypan blue exclusion method. The CFSE labeled T cell population was then cultured alone, or co-cultured with control and JEV infected NSPCs (3 dpi and without mitomycin C treatment) in supplemented RPMI media. Following co-culture for 72–80 hours, the cells were harvested and stained with CD3 antibody, and flow cytometry was performed.

### Flow Cytometry

Control and JEV infected neurospheres were dissociated into single cell suspension using Accutase treatment for 5–6 mins at 37°C with gentle shaking. The cells were washed with DMEM-F12, and then with staining buffer, counted and finally resuspended in FACS staining buffer (PBS with 3% FBS and 0.09% sodium azide). The cells were then stained with the desired antibodies, CD40, CD80, CD86, MHC class I (H-2k^d^) and rat IgG2a_κ_ isotype controls (BD Biosciences) for 45 mins on ice. Following washes with staining buffer, secondary antibody, anti-rat FITC was added for 30 mins on ice. The cells were finally washed and analyzed in FACS Calibur. For all conditions, 20,000 events were acquired and histograms for staining were plotted. Since neurospheres are a heterogeneous population of stem and progenitor cells, often multiple peaks are present in the histograms. The mean fluorescent intensity (MFI) was determined for each of the peaks in individual condition. The average MFI of all the peaks for a particular condition was calculated and plotted as representative graphs.

In co-culture experiments of NSPCs with CFSE labeled T-cells, the total cell population was resuspended in FACS staining buffer. Following incubation with anti-CD3ε antibody (a kind gift from Dr. Satyajit Rath, National Institute of Immunology, New Delhi) for 30 mins on ice, PE-labeled anti-rat secondary antibody was used for 30 mins on ice. After extensive washes with FACS buffer, the cells were acquired in FACS Calibur. Gates were set on live lymphocyte population and analyzed for CD3 on FL2 and CFSE on FL1 channel using Cell Quest Pro software.

### RNA Extraction and Quantitative Real Time-PCR (qRT-PCR)

Total cellular RNA was isolated from control and JEV infected neurospheres at different dpi using RNAeasy Mini Kit (Qiagen, Hamburg, Germany). Random hexamer primers were used for cDNA synthesis using Advantage RT-PCR (Clontech, Mountain View, CA). The forward and reverse primers for IL-2 (Genebank Acc No. NM_008366.2) were 5′-GCAACTGTGGTGGACTTTCTGA- 3′ and 5′-GAGGGCTTGTTGAGATGATGCT-3′ respectively. 500 ng of cDNA was used as a template for performing qRT-PCR using SYBR Green Supermix (Bio-Rad, Hercules, CA) on ABI Prism 7700 sequence detection system (Applied Biosystems, Foster City, CA). The conditions for real time PCR were as follows: 95°C for 3 min (1 cycle), 94°C for 20 s, 55°C for 30 s, and 72°C for 40 s (40cycles). The dissociation curves were generated to check for the specificity of primer annealing to the template. The real time PCR results were analyzed using the iCycler Thermal Cycler Software (Applied Biosystems, Foster City, CA) and normalized with those from 18S rRNA internal control.

### Statistical Analysis

Statistical analysis was performed on all experiments using paired two-tailed Student's t-test. A statistical p value of 0.05 was considered significant.

## Results

### Expression of Costimulatory Molecules and MHC Class I in SVZ during JE Progression

**Figure 1:**
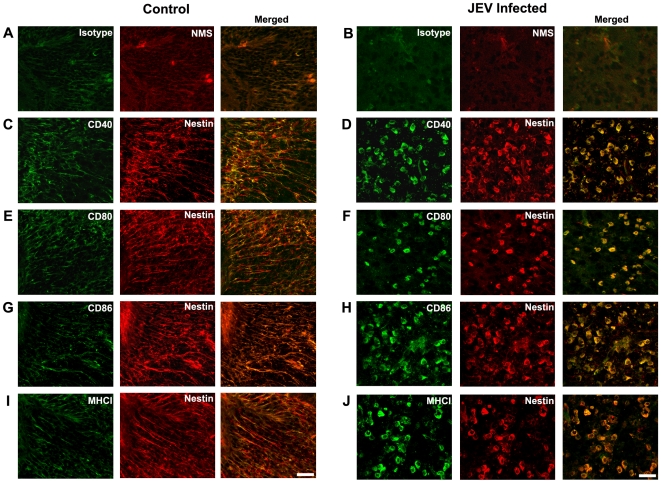
Upregulation of costimulatory molecules and MHC class I in Nestin-positive cells in SVZ during JEV infection. Brain cryosections from control and JEV infected animals were stained with antibodies against CD40, CD80, CD86, and MHC class I molecules and Nestin. Isotype staining using rat IgG2a_κ_ (for costimulatory molecules and MHC class I) and normal mouse serum (NMS; for Nestin) was performed on both control (**A**) and JEV infected brain sections (**B**). Co-localization of Nestin (red) with the costimulatory molecules and MHC class I (green) was performed using confocal microscopy. Isotype staining on both control and JEV infected sections show no detectable fluorescence (**A–B**). The Nestin +ve cells show a distinctive morphological change following infection from process bearing cells to round/oval shaped cells. Nestin positive cells in control sections show very less co-localization with all the costimulatory molecules and MHC class I (**C, E, G, I**). JEV infected SVZ show complete and increased co-localisation of Nestin positive cells with CD40 (**D**), CD80 (**F**), CD86 (**H**), and MHC class I (**J**). Scale bar corresponds to 20 microns.

We have previously reported that the infected SVZ harbours a high viral load of JEV compared to other brain areas. During the course of JEV infection, the Nestin positive (+) cells in the SVZ undergo morphological alterations as well as decrease in their cell number [25]. We therefore wanted to investigate the expression profile of different costimulatory molecules in the SVZ of infected animals. Immunohistochemistry was performed on control and JEV infected sections (4 dpi) for Nestin along with all the costimulatory molecules and MHC class I antigens. Nestin labels all cells in the SVZ with stem cell property, including neural stem cells, ependymal cells and neural progenitor/precursor cells. In control animals, Nestin + cells in the SVZ show elongated processes and very low expression of all the costimulatory molecules and MHC class I, as detected by dual immunofluorescence using confocal microscopy (Fig. 1 C, E, G, I). Following JEV infection, the expression of CD40, CD80, CD86 as well as MHC class I showed a prominent increase, and was completely colocalised with Nestin + cells in the SVZ (Fig. 1 D, F, H, J). The striking morphological change in Nestin + cells from cells with elongated process like structures to oval or round shaped cells was observed, which is in concordance with our earlier findings [25]. Furthermore, the expression of the costimulatory and MHC class I proteins on Nestin + cells in the JEV infected brain was mainly localised to the surface and partially to the cytoplasm as detected by co-localisation studies with the nuclear stain DAPI (Fig S1). These figures clearly indicate that the surface expression of costimulatory and MHC class I molecules underwent an increase in the Nestin positive cells in the SVZ of JEV infected animals.

### Expression of Costimulatory Molecules and MHC Class I Protein In Vitro by NSPCs during Progressive Infection

**Figure 2:**
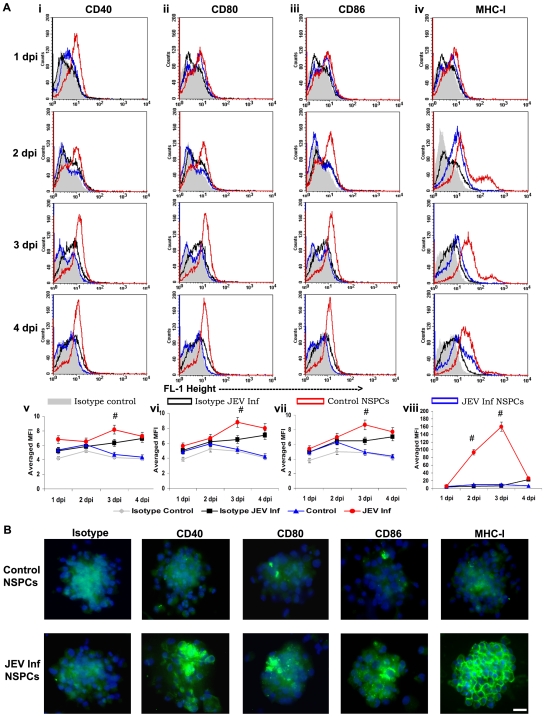
Increased surface expression of costimulatory molecules and MHC class I by NSPCs following JEV infection *in vitro*. NSPCs isolated from the SVZ were cultured as free-floating neurospheres and infected with JEV at MOI = 5. Following infection, the cells were cultured and collected at different time points from 1 dpi to 4 dpi. Control and JEV infected NSPCs were stained with anti-CD40, anti-CD80, anti-CD86, and anti-MHC class I and isotype controls (IgG2a_κ_) antibodies, and analyzed using BD FACS Calibur system. The histograms were plotted and the mean fluorescent intensity (MFI) was calculated over all the existing peaks in each of different conditions and finally represented graphically as the averaged MFI for each condition (**2A**). Significant increase in surface expression of CD40 (**2A** **i, v**), CD80 (**2A** **ii, vi**) and CD86 (**2A** **iii, viii**) was noted at 3 dpi in JEV infected NSPCs. MHC class I (**2A** **iv, viii**) expression was upregulated significantly at both 2 and 3 dpi in the infected NSPCs compared to control NSPCs and JEV infected isotype. # indicates p<0.05 compared to control NSPCs and infected isotypes. Control and JEV infected neurospheres at 3 dpi were collected and allowed to adhere on PDL-coated chamber slides for 3 hours and immunostained for CD40, CD80, CD 86 and MHC class I molecules, as well as rat IgG2a_κ_ isotype (**2B**). The nuclear counterstain was done with DAPI (blue). Increased surface expression of all the costimulatory molecules (CD40, 80, 86) was observed in infected neurospheres. Robust surface staining for MHC class I was noticed in the infected NSPCs compared to uninfected control. Scale bar corresponds to 20 microns.

NSPCs were isolated from SVZ of BALB/c mouse pups and cultured in serum free media supplemented with growth factors as free-floating clusters of cells called neurospheres. These spheres expressed nestin [25] and when cultured in differentiation media formed both neurons and astrocytes. After two passages, NSPCs were either uninfected or JEV infected (MOI = 5) and collected at 1 dpi, 2 dpi, 3 dpi and 4 dpi to evaluate progressive infection in them. Using flow cytometry, we determined the expression of both costimulatory and MHC class I molecules on both control (uninfected) and JEV infected NSPCs at different dpi. Control NSPCs showed negligible expression of costimulatory molecules, CD40, CD80, and CD86, comparable to isotype controls. Interestingly, with progressive JEV infection, the surface expression of all these molecules underwent a gradual increase with increasing dpi (Fig. 2A i–iv). The mean fluorescent intensity (MFI) for each of these surface costimulatory molecules was calculated as an average of the MFI from multiple peaks in all the 4 conditions- isotype control, isotype JEV infected, control neurospheres and JEV infected neurospheres. The graphs representing averaged MFI values show a similar pattern of expression for costimulatory and MHC class I molecules in infected NSPCs- a gradual increase till 3 dpi, and a sharp decline at 4 dpi (Fig. 2A v–viii). Significant upregulation of CD40, CD80 and CD86 was observed in JEV infected NSPCs at 3 dpi only compared to uninfected controls and JEV infected isotype controls (Fig. 2A i, ii, iii v, vi, vii) (p<0.05). MHC class I expression in the JEV infected NSPCs also demonstrated a progressive increase, with robust expression at 3 dpi, which declined drastically at 4 dpi. The expression of MHC class I on JEV infected NSPCs was elevated significantly over control NSPCs and infected isotype controls at 2 and 3 dpi (Fig. 2A iv, viii). MHC class II expression however remained unaltered post JEV infection (data not shown).

Furthermore, immunocytochemistry was performed on control and JEV infected (3 dpi) neurospheres for both costimulatory molecules and MHC class I antigens. Since flow cytometry revealed maximum expression of these molecules on 3 dpi of JEV infection, we have chosen this time point for immunocytochemistry to further validate the surface expression of immunological molecules by infected NSPCs (Fig. 2B). The JEV infected neurospheres showed high surface expression of the costimulatory molecules compared to control neurospheres and infected isotypes. The robust expression of MHC class I was also seen upon virus infection and was confined to the surface of these NSPCs.

### Induction of Different Cytokines in NSPCs upon Progressive JEV Infection

**Figure 3:**
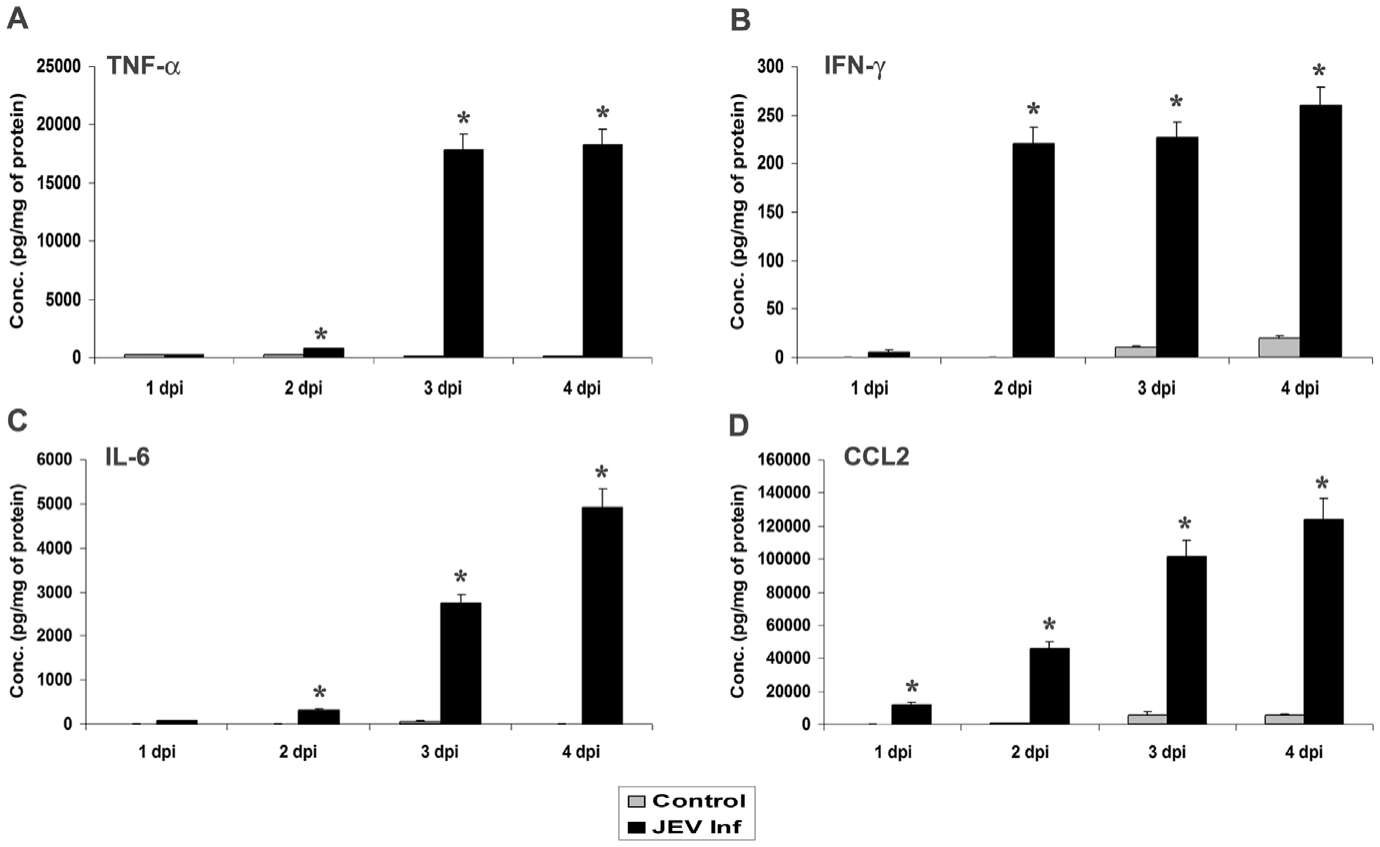
Production of inflammatory cytokines by NSPCs upon JEV infection. Control and JEV infected neurospheres were collected at 1–4 dpi and cell lysates were prepared. CBA was performed with the cell lysates and the graphs represent the concentration of the different cytokines produced. Significant production of TNF-α (**A**), IFN-γ (**B**), IL-6 (**C**), and CCL2/MCP-1 (**D**) was observed in JEV infected NSPCs compared to control NSPCs, and the levels of all cyto/chemokines reached the maximum at 3 dpi and 4 dpi. Values represent the means ± SEM from three independent experiments. * indicates p<0.005 compared to control.

Previous studies have reported that TNF-α induces the production of chemokines like CCL2 and IP-10 from human neural precursor cells [29]. It is also established that JEV infection leads to massive upregulation of inflammatory cytokine levels [19], and neurons also respond to infection by secreting CCL2, and type I and II IFNs [21]. We therefore hypothesized that virus infection in NSPCs would also trigger the production of various cyto/chemokines. To test the hypothesis, control and JEV infected neurospheres at different time points of infection (1 dpi–4 dpi) were collected and cell lysates were prepared from them. CBA was performed using the cell lysate and bead mixture and the production of different cytokines was assessed. We found a significant increase in TNF-α, IFN-γ, IL-6 and CCL-2 production in JEV infected neurospheres compared to lysates from uninfected control (Fig. 3) (p<0.005). While TNF-α, IFN-γ, and IL-6 levels showed significant elevation from 2 dpi onwards (Fig. 3 A–C) the level of chemokine CCL2 was up-regulated as early as 1 dpi and showed a progressive increase till 4 dpi of JEV infection (Fig. 3D). Thus, the production of all the cyto/chemokines increased multifold in infected NSPCs and possibly contribute to the development of inflammatory upheaval in the CNS following JEV infection.

### JEV Infected NSPCs Are Capable of Providing Functional Costimulation to T-Cells

**Figure 4:**
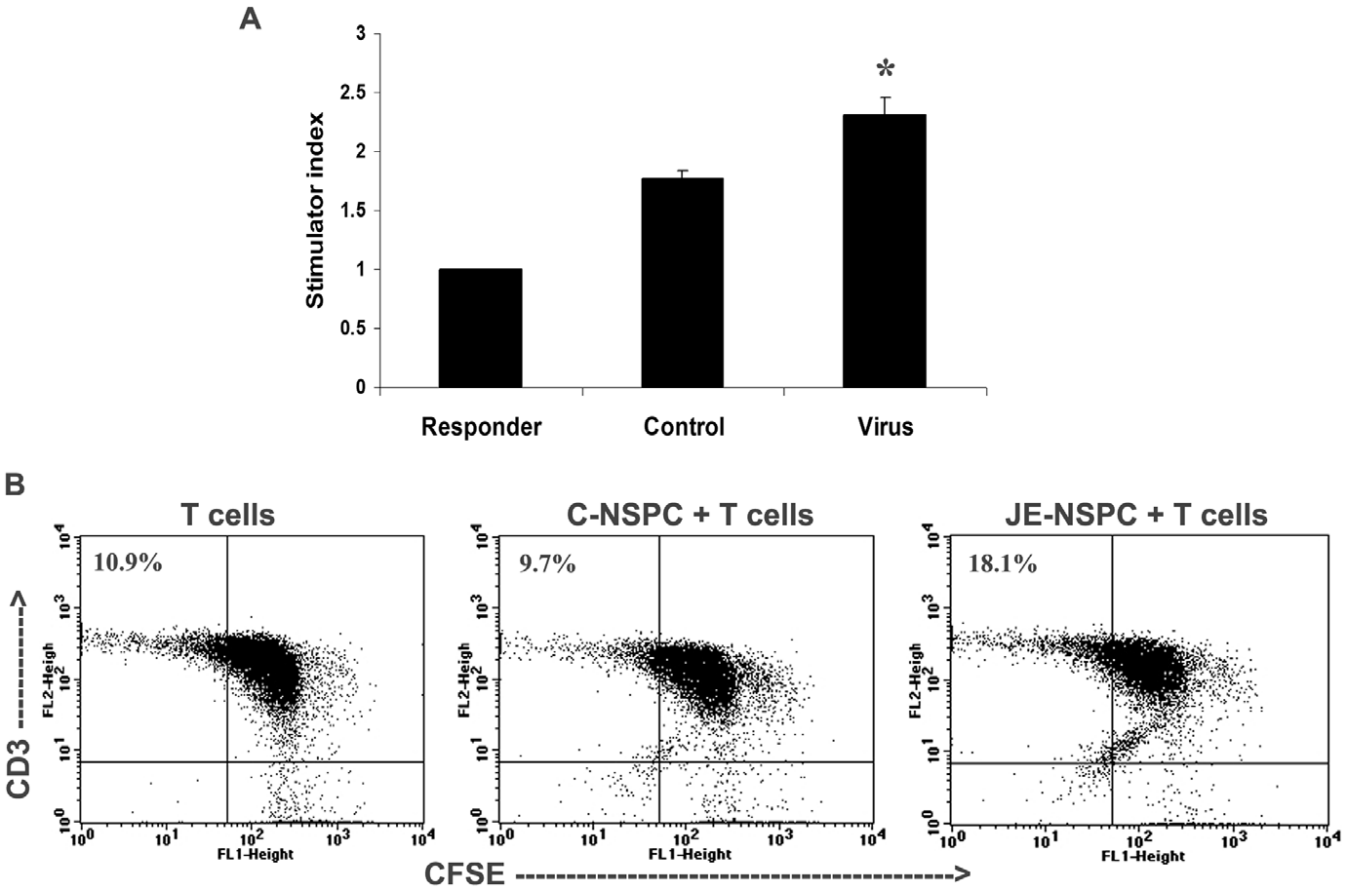
Stimulation of T cell proliferation by NSPCs upon JEV infection. Control and JEV infected NSPCs were dissociated into single cell suspension and treated with mitomycin C. Purified splenic T cells were used as responders and co-cultured with control and infected NSPCs (stimulators) for 72 hours. MTS reagent was used to detect proliferation and absorbance was measured at 490 nm. Experiments were performed in triplicate. The graph represents the stimulator index (SI), which shows that JEV infected NSPCs have a higher stimulator index, i.e. stimulate greater T cell proliferation compared to control NSPCs (**A**). * indicates p<0.05 compared to control. Purified splenic T cells were labeled with CFSE and then cultured alone or co-cultured with control and JEV infected NSPCs for 72–80 hours. The cells were then stained for anti-CD3ε antibody and analyzed by FACS. The dot plots represent the population of CD3 detected on FL2 channel and CFSE label on FL1 channel. The decrease of CFSE staining is an indication of proliferation and we quantified the percentage of CD3 +ve cells, which have low CFSE label (upper left quadrant of dot plots) (**B**). JEV infected NSPCs induced T cell proliferation by 2 fold compared to control NSPCs (p<0.01).

Since high surface expression of CD40, CD80 and CD86 along with MHC class I was observed in NSPCs upon JEV infection, it was important to establish the functional significance of such expression. The ability of NSPCs to interact with allogenic T-cells has already been shown and the NSPC:T cell conjugate formation has been favored upon stimulation of NSPCs with IFN- γ, i.e. in an inflammatory environment [13]. We therefore tested whether JEV infected NSPCs were able to provide functional costimulation to allogenic T cells. Control and JEV infected NSPCs at 3 dpi were collected and treated with mitomycin C, which arrests their cell cycle and growth. Mitomycin C treated control and infected NSPCs were used as stimulators in an allogenic mixed lymphocyte culture with purified T cells from C57/BL6 as responders. In a control experiment, the purified T cells were cultured in supernatant obtained from virus infected NSPCs, to rule out the effect of the viral antigen on proliferation of T cells (data not shown). The stimulator index was calculated as described before, and that of T cells was considered as 1. Co-culture with JEV infected NSPCs yielded a significantly higher stimulation index for T cells compared to co-culture with control NSPCs (Fig. 4A) (p<0.05). In other words, JEV infected NSPCs were able to stimulate T-cell proliferation significantly compared to control NSPCs where minimal T cell proliferation was observed.

The proliferative response of T cells in an allogenic reaction was further confirmed by performing CFSE labeling experiments. Purified T cells from C57/BL6 mice were CFSE labeled and either cultured alone or co-cultured with NSPCs as stimulators for 72–80 hours. The total cell population was collected and then stained with anti-CD3ε antibody and subjected to flow cytometry. On a live lymphocyte gate, the decrease of CFSE label in the CD3+ T cell population was studied (Fig. 4B). The CD3+/CFSE low population (upper left quadrant of dot plot) was quantified and we found 9.7±2.2% of T cells undergoing proliferating when co-cultured with control neurospheres. Interestingly, co-culture with JEV infected neurospheres increased the percentage of these proliferating T-cells (CD3+/CFSE low) to 18.1±2.96%, almost a 2 fold induction in proliferation (p<0.01). Thus, JEV infected NSPCs induced the proliferation of allogenic T cells significantly over control NSPCs, establishing the ability of the infected NSPCs to provide functional costimulation to T cells.

### Expression of IL-2 mRNA by NSPCs upon JEV Infection

**Figure 5:**
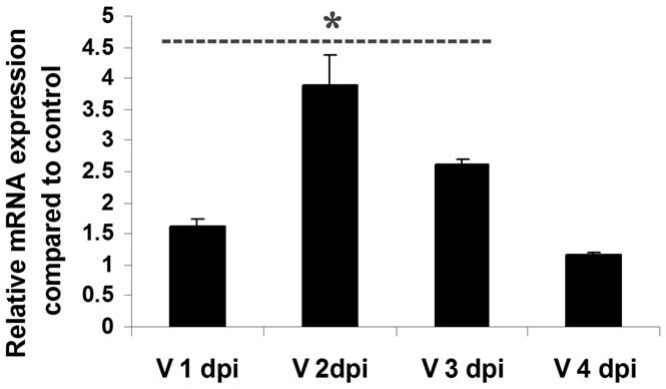
Induction of IL-2 mRNA expression by NSPCs upon JEV infection. RNA was isolated from control and JEV infected NSPCs at different dpi and cDNA was prepared from them. qRT-PCR was performed for IL-2 mRNA and normalised to 18S rRNA internal control. The graph represents the relative expression of IL-2 in JEV infected NSPCs at different dpi compared to control NSPCs. A significant increase in IL-2 mRNA levels was observed in JEV infected NSPCs from 1 dpi to 3 dpi compared to control NSPCs. * indicates p<0.01 compared to control.

Previous reports have highlighted the role of NSPCs in immunomodulation and regulation of Th1/Th2 balance by secretion of various immunomodulatory cytokines like TGF-β [18]. However, no evidence exists regarding the expression of cytokines like IL-2 which may influence T cell growth and proliferation. qRT-PCR was performed for detecting IL-2 mRNA expression using cDNA from control and JEV infected NSPCs at different time points of infection, from 1 dpi to 4 dpi. Interestingly, we observed that the NSPCs were capable of expressing IL-2 mRNA, whose levels underwent a significant increase upon JEV infection (Fig. 5) (p<0.01). The maximum increase in mRNA levels was noted at 2 dpi and 3 dpi of JEV infection. The expression of IL-2 by NSPCs possibly indicates a role of these cells in modulating the proliferation of T-cells. The enhanced IL-2 production upon JEV infection, therefore implicate that NSPCs trigger T cell proliferation and may contribute to the adaptive immune axis in the CNS.

## Discussion

The CNS has long been regarded as a privileged site that is exempted from immune surveillance, mainly due to the presence of physical barriers, like the blood - brain barrier and the blood-CSF barrier, and the virtual absence of CNS lymphatic system. However, increasing evidences have challenged this view and has supported the existence of an innate immune system within the brain. Besides the role of glial cells, mainly microglia and astrocytes in evoking an innate immune response to any pathogen, the neural elements also respond to pathogen attack in unique ways. Recent evidence from transplantation medicine have strongly supported that NSPCs interact with the immune system, by expression of various immune-relevant molecules. NSPCs have been shown to express cell adhesion molecules, integrins and chemokine receptors that allow them to interact with an inflamed CNS microenvironment [30].

In this study we have investigated the immunological properties of NSPCs following neurotropic viral infection (JEV infection in our case) and how these NSPCs interact with the different immune axes. A number of RNA viruses invade the CNS and infect the NSPCs to establish either lytic or latent infection in these cells. Here, we show for the first time that virus infection triggers immunogenicity in NSPCs and the possible mechanisms by which these infected NSPCs may contribute to the generation of host immune response.

To assess the immunogenic potential of virus infected NSPCs, we have determined the production of different cytokines and chemokines by these NSPCs using Cytokine Bead Array. Indeed, JEV infection in NSPCs increased the production of TNF-α, IFN-γ, IL-6 and CCL2. Both TNF-α and IFN-γ are prototypical Th1 cytokines, whose expression are linked to macrophage activation and subsequent inflammatory processes. Moreover, rapid local production of IFN-γ is an important survival strategy in most virus infections of the CNS. This type II interferon contributes to the innate immune response of the host by slowing viral spread and limiting viral replication before the induction of specific adaptive immune response [21]. The other cytokine IL-6 is a pleiotropic cytokine that promotes astrocyte proliferation and astrogliosis, as well as activation of brain macrophages [31]. JEV infected NSPCs also secrete the chemokine CCL2, a potent monocyte chemoattractant that signals to peripheral blood monocytes and activated T cells which express its receptor CCR2 [32], [33]. Thus, expression of IL-6 and CCL2 by JEV infected NSPCs aid in the recruitment of monocytes and activated T cells into the CNS, and thereby mount an immunological response against the virus.

We have previously reported that during the course of JEV infection, the SVZ is damaged and the NSPC pool is depleted, eventually leading to a deficit in CNS repair and regeneration. The infection of NSPC pool in the SVZ results in morphological alterations and proliferation arrest of this putative stem cell population, which may culminate in impaired neurogenesis [25]. In an experimental model of JEV infection, we have observed that the Nestin positive cells express and up-regulate the expression of costimulatory molecules, CD40, CD80 and CD86 on their surface. Nestin typically is a marker for both neural stem and/or progenitor cells lying in the neurogenic niches of the brain. In neurosphere cultures also, which constitute a heterogeneous population of neural stem and progenitor cells, we have observed that with progressive JEV infection, there is a significant increase in expression of CD40 and both B7 molecules. Significant expression of all these costimulatory molecules was noted at 3 dpi, the time point which we presume when maximum viral load is present in these NSPCs. Previous reports have indicated expression of costimulatory molecules in the CNS following viral infection, mainly by astrocytes and microglia, which are the resident immune cells of the CNS [31], [34]. Moreover, it has been reported that costimulatory molecules are also expressed by cells which are not considered professional Antigen Presenting Cells (APC) like neurons [35], fibroblasts [36] etc. Therefore, the expression of all the costimulatory molecules indicates that the NSPCs might have some role in non-professional antigen presentation following viral invasion.

There has been lot of controversy regarding MHC expression in NSPCs, and earlier most studies have demonstrated low surface expression of both MHC class I and class II. Recently, however, MHC class I expression in NSPCs from mouse, rat and humans has been documented, and both murine and human NSPCs are able to trigger an allogenic reaction and stimulate the proliferation of allogenic T-cells [13], [15]. We also show upon JEV infection, the surface expression of MHC class I (but not class II) on NSPCs increased robustly at 2 and 3 dpi *in vitro*. High expression of MHC class I on the Nestin positive cells in the SVZ of infected animals was also observed. Our observations support previous studies showing induction of both classical and non-classical MHC class I antigens after JEV infection, but not of MHC class II [37]. The immune properties of NSPCs are very similar to those of neurons, which also rarely express MHC class I molecules [38], but may be induced to express high levels of MHC class I after axotomy or lesions, treatment with cytokines, and viral infections [39], [40], [41]. MHC class I signaling has been shown to be important for synaptic plasticity during normal development as well as during axonal regeneration following axotomy or lesions [41], [42]. Therefore, the high expression of MHC class I by the infected NSPCs, may suggest a role of MHC signaling in these cells to aid in functional recovery from viral infection.

We further demonstrate that high costimulatory molecule expression on virus infected neurospheres is immunologically functional, i.e. JEV infected NSPCs are able to costimulate T cells. Both Mixed Lymphocyte reaction and CFSE experiments have validated that co-culture of allogenic T cells with JEV infected NSPCs can stimulate T-cell proliferation significantly compared to co-culture with uninfected NSPCs. Besides providing functional costimulation, the virus infected neurospheres also provide mitogenic signals for T-cell proliferation and activation. IL-2, a Th1 cytokine induces clonal expansion of T cells during an immune response without determining the antigen specificity [43]. Though previous reports have shown no detectable expression of IL-2 mRNA in human NSPCs even after stimulation with IFN-γ and TNF-α, we observed conspicuous induction of IL-2 mRNA in mouse NSPCs cells by qRT-PCR. JEV infection in NSPCs induced the expression of IL-2 mRNA, which reached its peak at 2–3 dpi. Thus, it can be clearly stated that NSPCs stimulate the adaptive immune response by production of Th1 cytokines, which in turn regulate the proliferation of T cells.

To summarize, murine NSPCs may respond to neurotropic virus infection by:

- i) Production of different cytokines and chemokines like IFN-γ and IL-6, which activate the prototypical immune cells of the brain, mainly astrocytes and microglia. IFN-γ also contributes to the generation of an innate immune response to viral infection. Soluble mediators released from infected NSPCs activate microglia and stimulate production of cytochemokines like TNF-α, IL-6, IFN-γ and CCL2 (unpublished observations).
- ii) Certain cyto/chemokines like TNF-α and CCL2 increase the expression of cell adhesion molecules on endothelial cells and aid in infiltration of peripheral T-cells and monocytes by crossing the Blood Brain Barrier.
- iii) Uupregulation of costimulatory molecules and MHC class I expression upon virus infection on NSPCs allows these cells to effectively costimulate T cells and also trigger their proliferation (via IL-2 production). Thus, the NSPCs may also act on the adaptive immune axis and help in generation of antigen/virus specific immune response.

**Figure 6:**
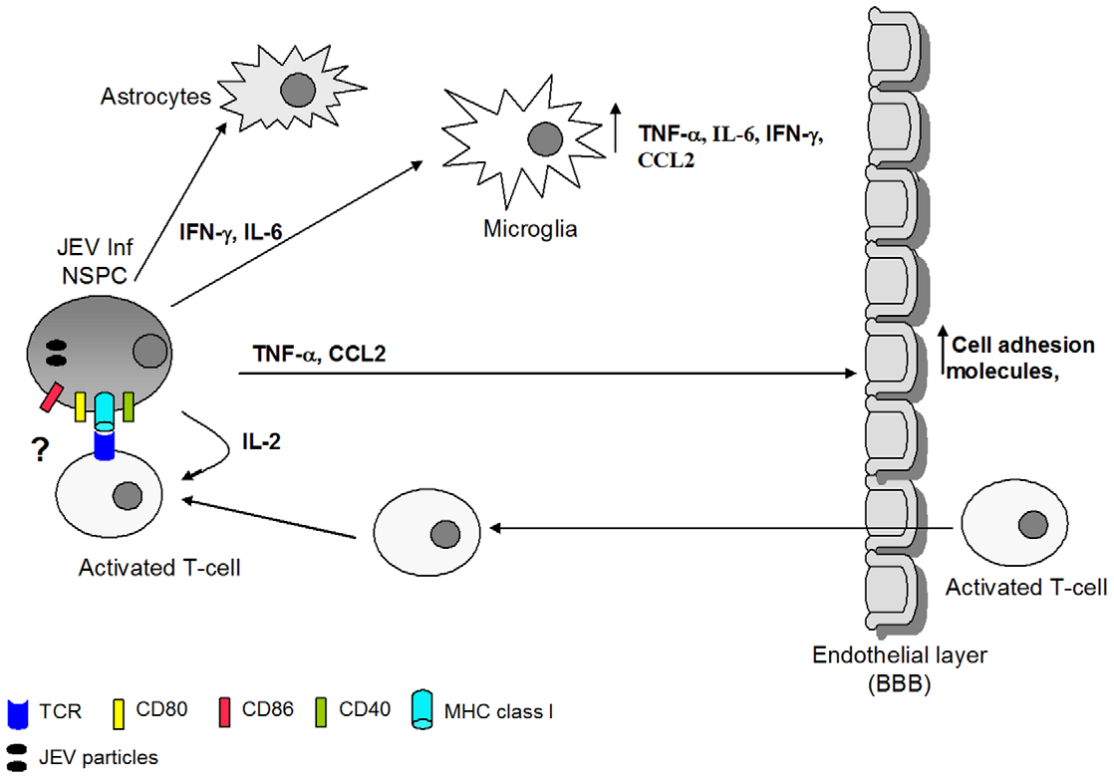
Schematic representation of the possible role of NSPCs in induction of an immune response to virus infection. Following JEV infection, NSPCs secrete an array of cyto/chemokines which help in the generation of an innate immune response against the viral pathogen. IFN-γ and IL-6 released form JEV infected NSPCs serve to activate the astrocytes and microglia, the primary immune cells of the CNS. Furthermore, both TNF-α and CCL2 have proven roles in upregulation of cell adhesion molecules on the endothelial cells of the blood brain barrier. These cell adhesion molecules help in recruitment of the activated T cells and monocytes from the periphery, which can now easily enter the CNS. On the other hand, JEV infection leads to upregulation of surface expression of costimulatory molecules like CD40, CD80, CD86 as well as MHC class I antigens on NSPCs. These costimulatory molecules and MHC class I on NSPCs are capable of providing functional co-stimulation to T cells and promote their proliferation. Furthermore, infected NSPCs also produce IL-2 which provides mitogenic signals for T cell proliferation. Thus, infected NSPCs are capable of stimulating the adaptive immune system, but whether the formation of an immunological synapse with T cells occurs is still under investigation.

Here, we have identified the immunological properties of NSPCs in response to virus infection, and have proposed a model describing how these infected NSPCs may modulate both the innate and adaptive immune system in the CNS (Fig. 6). Recently it has been shown that NSPCs modulate T cell mediated immune response by release of various soluble mediators [28]. We are unable to conclusively state at this point whether the infected NSPCs trigger the proliferation of CD4+ or CD8+ T cells, and future work is underway to identify the responding T cell population. Existing reports show the formation of an immunological synapse between the NSPC and T cell, and how the formation increases in an inflammatory environment [13]. The ability of infected NSPCs to form a functional immunological synapse with T cells and cross-present viral antigens also needs to be deciphered. Further insights about the immuno-competency of these cells in combating any infection or insult will help to identify a new function of these cells in non-professional antigen presentation in the injured CNS. The immunological role played by NSPCs in RNA virus infections has been proven, but their interaction with the other immune components both in the CNS and in the periphery needs to be further elucidated.

## Acknowledgments

The authors thank Mr. R. Khader Valli for help in qRT-PCR experiments.

## Supporting Information

Figure S1 Surface and cytoplasmic expression of costimulatory molecules and MHC class I in Nestin-positive cells in SVZ during JEV infection. Cryosections from brains of JEV infected animals were stained with antibodies against CD40, CD80, CD86, and MHC class I molecules and Nestin. Images from the SVZ were captured using 40x oil immersion lens in Zeiss Apotome microscope. DAPI was used as the nuclear counterstain. Merged images represent Nestin positive cells (red) co-localized with CD40 (A), CD80 (B), CD86 (C), and MHC class I (D) (green) along with nuclear stain DAPI (blue). The expression of all the costimulatory molecules and MHC class I is localised primarily to the surface and/or cytoplasm of the Nestin positive cells in the JEV infected SVZ. Scale bar corresponds to 20 microns.
(1.10 MB TIF)

## References

[1] Romanko MJ, Rola R, Fike JR, Szele FG, Dizon ML (2004). Roles of the mammalian subventricular zone in cell replacement after brain injury.. Prog Neurobiol.

[2] Park KI, Teng YD, Snyder EY (2002). The injured brain interacts reciprocally with neural stem cells supported by scaffolds to reconstitute lost tissue.. Nat Biotechnol.

[3] Ming GL, Song H (2005). Adult neurogenesis in the mammalian central nervous system.. Annu Rev Neurosci.

[4] Kelly S, Bliss TM, Shah AK, Sun GH, Ma M (2004). Transplanted human fetal neural stem cells survive, migrate, and differentiate in ischemic rat cerebral cortex.. Proc Natl Acad Sci U S A.

[5] Ourednik J, Ourednik V, Lynch WP, Schachner M, Snyder EY (2002). Neural stem cells display an inherent mechanism for rescuing dysfunctional neurons.. Nat Biotechnol.

[6] Cummings BJ, Uchida N, Tamaki SJ, Salazar DL, Hooshmand M (2005). Human neural stem cells differentiate and promote locomotor recovery in spinal cord-injured mice.. Proc Natl Acad Sci U S A.

[7] Einstein O, Ben-Hur T (2008). The changing face of neural stem cell therapy in neurologic diseases.. Arch Neurol.

[8] Pluchino S, Zanotti L, Deleidi M, Martino G (2005). Neural stem cells and their use as therapeutic tool in neurological disorders.. Brain Res Brain Res Rev.

[9] Mammolenti M, Gajavelli S, Tsoulfas P, Levy R (2004). Absence of major histocompatibility complex class I on neural stem cells does not permit natural killer cell killing and prevents recognition by alloreactive cytotoxic T lymphocytes in vitro.. Stem Cells.

[10] Odeberg J, Piao JH, Samuelsson EB, Falci S, Akesson E (2005). Low immunogenicity of in vitro-expanded human neural cells despite high MHC expression.. J Neuroimmunol.

[11] Hori J, Ng TF, Shatos M, Klassen H, Streilein JW (2003). Neural progenitor cells lack immunogenicity and resist destruction as allografts.. Stem Cells.

[12] Hori J, Ng TF, Shatos M, Klassen H, Streilein JW (2007). Neural progenitor cells lack immunogenicity and resist destruction as allografts. 2003.. Ocul Immunol Inflamm.

[13] Imitola J, Comabella M, Chandraker AK, Dangond F, Sayegh MH (2004). Neural stem/progenitor cells express costimulatory molecules that are differentially regulated by inflammatory and apoptotic stimuli.. Am J Pathol.

[14] Johansson S, Price J, Modo M (2008). Effect of inflammatory cytokines on major histocompatibility complex expression and differentiation of human neural stem/progenitor cells.. Stem Cells.

[15] Ubiali F, Nava S, Nessi V, Frigerio S, Parati E (2007). Allorecognition of human neural stem cells by peripheral blood lymphocytes despite low expression of MHC molecules: role of TGF-beta in modulating proliferation.. Int Immunol.

[16] Pluchino S, Zanotti L, Rossi B, Brambilla E, Ottoboni L (2005). Neurosphere-derived multipotent precursors promote neuroprotection by an immunomodulatory mechanism.. Nature.

[17] Einstein O, Fainstein N, Vaknin I, Mizrachi-Kol R, Reihartz E (2007). Neural precursors attenuate autoimmune encephalomyelitis by peripheral immunosuppression.. Ann Neurol.

[18] Ben-Hur T (2008). Immunomodulation by neural stem cells.. J Neurol Sci.

[19] Ghoshal A, Das S, Ghosh S, Mishra MK, Sharma V (2007). Proinflammatory mediators released by activated microglia induces neuronal death in Japanese encephalitis.. Glia.

[20] Bhowmick S, Duseja R, Das S, Appaiahgiri MB, Vrati S (2007). Induction of IP-10 (CXCL10) in astrocytes following Japanese encephalitis.. Neurosci Lett.

[21] Griffin DE (2003). Immune responses to RNA-virus infections of the CNS.. Nat Rev Immunol.

[22] Neumann H (2001). Control of glial immune function by neurons.. Glia.

[23] Vrati S, Agarwal V, Malik P, Wani SA, Saini M (1999). Molecular characterization of an Indian isolate of Japanese encephalitis virus that shows an extended lag phase during growth.. J Gen Virol.

[24] Dutta K, Ghosh D, Basu A (2009). Curcumin protects neuronal cells from Japanese encephalitis virus-mediated cell death and also inhibits infective viral particle formation by dysregulation of ubiquitin-proteasome system.. J Neuroimmune Pharmacol.

[25] Das S, Basu A (2008). Japanese encephalitis virus infects neural progenitor cells and decreases their proliferation.. J Neurochem.

[26] Swarup V, Das S, Ghosh S, Basu A (2007). Tumor necrosis factor receptor-1-induced neuronal death by TRADD contributes to the pathogenesis of Japanese encephalitis.. J Neurochem.

[27] Tai P, Wang J, Jin H, Song X, Yan J (2008). Induction of regulatory T cells by physiological level estrogen.. J Cell Physiol.

[28] Kim SY, Cho HS, Yang SH, Shin JY, Kim JS (2009). Soluble mediators from human neural stem cells play a critical role in suppression of T-cell activation and proliferation.. J Neurosci Res.

[29] Sheng WS, Hu S, Ni HT, Rowen TN, Lokensgard JR (2005). TNF-alpha-induced chemokine production and apoptosis in human neural precursor cells.. J Leukoc Biol.

[30] Martino G, Pluchino S (2007). Neural stem cells: guardians of the brain.. Nat Cell Biol.

[31] Dong Y, Benveniste EN (2001). Immune function of astrocytes.. Glia.

[32] Gendelman HE, Ding S, Gong N, Liu J, Ramirez SH (2009). Monocyte chemotactic protein-1 regulates voltage-gated K+ channels and macrophage transmigration.. J Neuroimmune Pharmacol.

[33] Taub DD, Proost P, Murphy WJ, Anver M, Longo DL (1995). Monocyte chemotactic protein-1 (MCP-1), -2, and -3 are chemotactic for human T lymphocytes.. J Clin Invest.

[34] Aloisi F (2001). Immune function of microglia.. Glia.

[35] Issazadeh S, Navikas V, Schaub M, Sayegh M, Khoury S (1998). Kinetics of expression of costimulatory molecules and their ligands in murine relapsing experimental autoimmune encephalomyelitis in vivo.. J Immunol.

[36] Pechhold K, Patterson NB, Craighead N, Lee KP, June CH (1997). Inflammatory cytokines IFN-gamma plus TNF-alpha induce regulated expression of CD80 (B7-1) but not CD86 (B7-2) on murine fibroblasts.. J Immunol.

[37] Abraham S, Manjunath R (2006). Induction of classical and nonclassical MHC-I on mouse brain astrocytes by Japanese encephalitis virus.. Virus Res.

[38] Miralves J, Magdeleine E, Kaddoum L, Brun H, Peries S (2007). High levels of MeCP2 depress MHC class I expression in neuronal cells.. PLoS One.

[39] Neumann H, Cavalie A, Jenne DE, Wekerle H (1995). Induction of MHC class I genes in neurons.. Science.

[40] Foster JA, Quan N, Stern EL, Kristensson K, Herkenham M (2002). Induced neuronal expression of class I major histocompatibility complex mRNA in acute and chronic inflammation models.. J Neuroimmunol.

[41] Thams S, Oliveira A, Cullheim S (2008). MHC class I expression and synaptic plasticity after nerve lesion.. Brain Res Rev.

[42] Huh GS, Boulanger LM, Du H, Riquelme PA, Brotz TM (2000). Functional requirement for class I MHC in CNS development and plasticity.. Science.

[43] Abbas AK (2003). The control of T cell activation vs. tolerance.. Autoimmun Rev.

