# Supplementary figures and images for: Japanese Encephalitis Virus Induce Immuno-Competency in Neural Stem/Progenitor Cells
Source: PLoS One. 2009 Dec 2;4(12):e8134. doi: 10.1371/journal.pone.0008134 (PMC2780913; doi:10.1371/journal.pone.0008134)

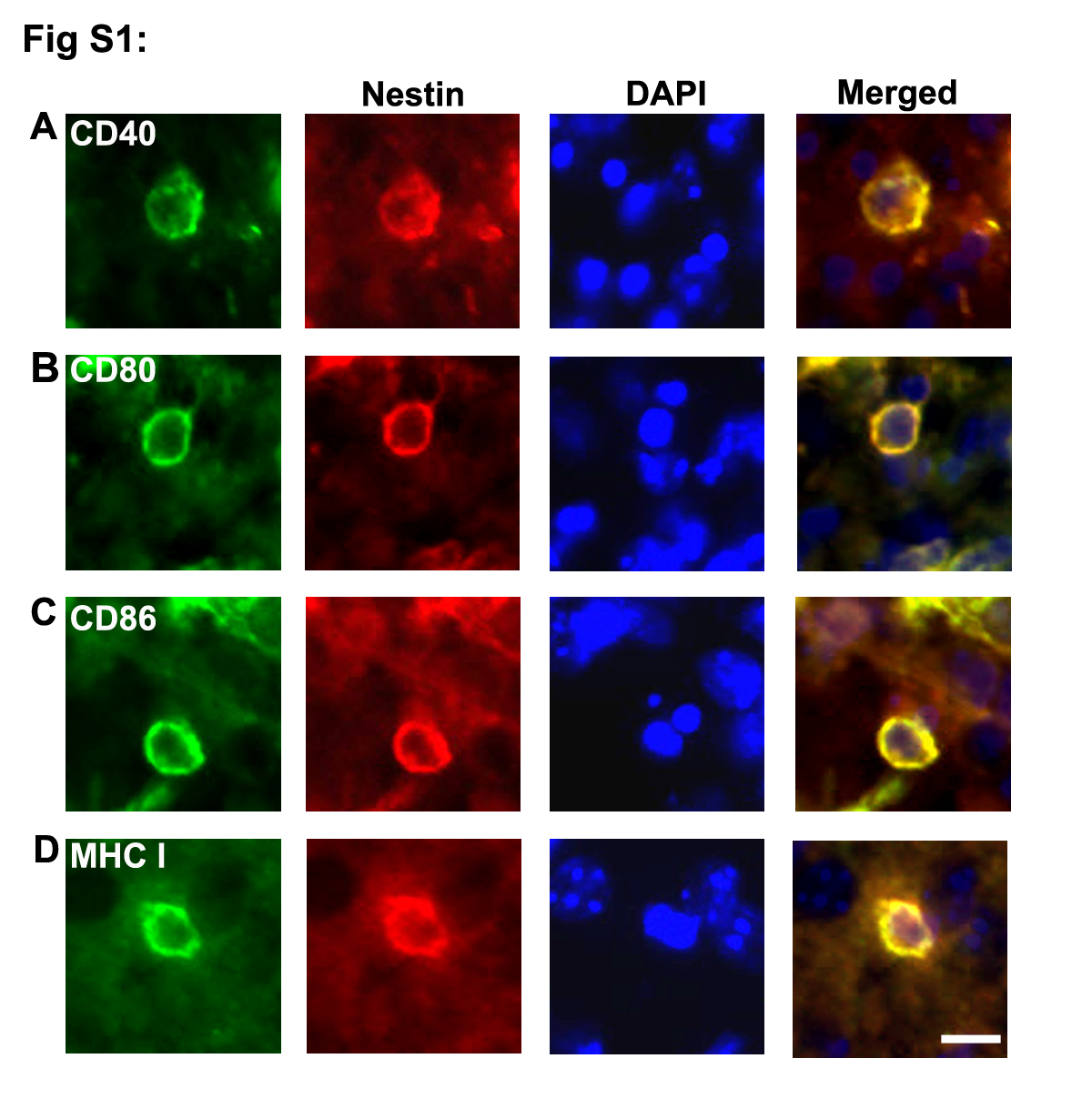

Supplement: Figure S1 — Surface and cytoplasmic expression of costimulatory molecules and MHC class I in Nestin-positive cells in SVZ during JEV infection. Cryosections from brains of JEV infected animals were stained with antibodies against CD40, CD80, CD86, and MHC class I molecules and Nestin. Images from the SVZ were captured using 40x oil immersion lens in Zeiss Apotome microscope. DAPI was used as the nuclear counterstain. Merged images represent Nestin positive cells (red) co-localized with CD40 (A), CD80 (B), CD86 (C), and MHC class I (D) (green) along with nuclear stain DAPI (blue). The expression of all the costimulatory molecules and MHC class I is localised primarily to the surface and/or cytoplasm of the Nestin positive cells in the JEV infected SVZ. Scale bar corresponds to 20 microns. (1.10 MB TIF) [file pone.0008134.s001.tif]
